# CafeteriaFCD Corpus: Food Consumption Data Annotated with Regard to Different Food Semantic Resources

**DOI:** 10.3390/foods11172684

**Published:** 2022-09-02

**Authors:** Gordana Ispirova, Gjorgjina Cenikj, Matevž Ogrinc, Eva Valenčič, Riste Stojanov, Peter Korošec, Ermanno Cavalli, Barbara Koroušić Seljak, Tome Eftimov

**Affiliations:** 1 Computer Systems Department, Jožef Stefan Institute, 1000 Ljubljana, Slovenia; 2 Jožef Stefan International Postgraduate School, 1000 Ljubljana, Slovenia; 3 School of Health Sciences, College of Health, Medicine and Wellbeing, University of Newcastle, Callaghan, NSW 2308, Australia; 4 Food and Nutrition Program, Hunter Medical Research Institute, Newcastle, NSW 2305, Australia; 5 Faculty of Computer Science and Engineering, “Ss. Cyril and Methodius” University in Skopje, 1000 Skopje, North Macedonia; 6 Resources and Support Department, European Food Safety Authority, 43126 Parma, Italy; 7 Faculty of Computer and Information Science, University of Ljubljana, 1000 Ljubljana, Slovenia

**Keywords:** food consumption data, recipe data, annotated corpus, semantic resource, semantic tags, annotation methods, gold standard corpus

## Abstract

Besides the numerous studies in the last decade involving food and nutrition data, this domain remains low resourced. Annotated corpuses are very useful tools for researchers and experts of the domain in question, as well as for data scientists for analysis. In this paper, we present the annotation process of food consumption data (recipes) with semantic tags from different semantic resources—Hansard taxonomy, FoodOn ontology, SNOMED CT terminology and the FoodEx2 classification system. FoodBase is an annotated corpus of food entities—recipes—which includes a curated version of 1000 instances, considered a gold standard. In this study, we use the curated version of FoodBase and two different approaches for annotating—the NCBO annotator (for the FoodOn and SNOMED CT annotations) and the semi-automatic StandFood method (for the FoodEx2 annotations). The end result is a new version of the golden standard of the FoodBase corpus, called the CafeteriaFCD (Cafeteria Food Consumption Data) corpus. This corpus contains food consumption data—recipes—annotated with semantic tags from the aforementioned four different external semantic resources. With these annotations, data interoperability is achieved between five semantic resources from different domains. This resource can be further utilized for developing and training different information extraction pipelines using state-of-the-art NLP approaches for tracing knowledge about food safety applications.

## 1. Introduction

In the past two decades, a large amount of work has been conducted to address information extraction (IE) in the biomedical domain. All of this work is supported by the existence of diverse biomedical vocabularies and standards, such as the Unified Medical Language System (UMLS), together with the collection of a large amount of annotated biomedical data (e.g., in the domain of drugs, diseases and other treatments) from numerous biomedical NLP workshops. The existence of such resources and IE methods allows exploring and investigating the biomedical domain and clinical practices. Conversely, the food domain still remains low resourced, which brings to attention the problem of developing new methods for the automated IE of food-related entities. There are very few semantic resources (i.e., FoodON [1], SNOMED CT [2] and Hansard taxonomy [3]) for food and nutrition-related data. This scarcity of semantic resources implies that IE methods for food and nutrition-related data will also be rare and limited, because they are highly dependent on these semantic external resources, as they are indispensable for building named-entity recognition (NER) methods and IE methods in general. In our domain of interest, i.e., the food and nutrition domain, there are a few IE methods available, more specifically rule-based methods for food IE—drNER [4] and FoodIe [5]. DrNER is a named-entity recognition system that uses rules to extract data from dietary recommendations that are supported by research. In addition to nutritional data, food concepts are also within the scope of this NER system. FoodIe is an expansion of this technique into an NER system with rules that are specifically designed for extracting food-related information. It is built around a rule engine, and the rules are based on semantic data that describe each culinary concept and computational linguistics. Despite these, there are no other NER methods specifically for the IE of food concepts. In order to build more resources like these, whether it be rule-based, dictionary-based or corpus-based NER, we need external semantic resources, which further need to be sustainable—meaning these resources need to be linked and interconnected—so that interoperability between them is enabled.

For this purpose, in 2019, we developed FoodBase, an annotated corpus of food entities, along with their normalization, using several food semantic resources [6]. To go in this direction, as a part of a European Food Safety Authority-funded project, we have extended this work and annotated the FoodBase corpus [6] across different semantic resources.

In Section 2, we provide the materials utilized in our study and the methodology we used to annotate the food consumption data with regard to different semantic resources. In Section 3, we provide the results—the annotated corpus across Hasnard, FoodOn, SNOMED CT [2] and FoodEx2. Finally, in Section 4, we conclude the paper with a discussion.

## 2. Materials and Methods

Here, we describe in detail the semantic resources used to annotate the food consumption data and create the CafeteriaFCD corpus. We start with the Hansard corpus of semantic tags [3,7], then we continue with describing three ontologies, available at the BioPortal [8]: FoodOn [1,9], OntoFood [10] and SNOMED CT [2], finally ending up with FoodEx2 [11] classification system. Next, a previously published food consumption annotated corpus with food entities called FoodBase [6] is explained, followed by a semantic resource called FoodOntoMap [12] that allows linking food concepts between different semantic resources. Further several methods utilized in our study are described in more detail: the NCBO annotator [13] can be used to obtain semantic tags for food entities with regard to ontologies that exist in the BioPortal [8], and the StandFood [14] method used for obtaining the FoodEx2 [11] codes for food entities. Finally, the main pipeline for annotating the food consumption data with regard to different semantic resources is explained in more detail.

### 2.1. Biomedical and Food Semantic Resources

#### 2.1.1. Hansard Taxonomy

This Hansard taxonomy, i.e., corpus [3,7], is a collection of texts which comprises practically every speech delivered in the British Parliament from 1803 to 2005. It comes to around 1.6 billion words in total, and it allows you to conduct semantically based searches on these speeches in ways that no other resource can. The corpus was developed as part of the UK Arts and Humanities Research Council-funded SAMUELS project (2014–2016). On each of the texts in the collection, lemmatization, part-of-speech tagging, and semantic tagging are performed. In [3,15], there is detailed information about the semantic tags included in the Hansard corpus. The semantic tags in the corpus are arranged using a hierarchical framework, and out of our interest for this study (because the semantic tags from the Hansard corpus are employed to annotate food concepts) is the ‘Food and drink’ semantic grouping with the semantic tag (AG). The ‘Food and drink’ category is one of the 37 upper-level semantic groupings. ‘Food’ (AG:01), ‘Production of food, farming’ (AG:02) and ‘Acquisition of animals for food, hunting’ (AG:03) are the three subcategories of the AG category. There are 125 top-level semantic tags in the ‘Food’ subcategory, 36 top level semantic tags in the ‘Production of food, farming’ subcategory, and 13 top-level semantic tags in the ‘Acquisition of animals for food, hunting’ subcategory. However, because there are some food concepts that are considered part of an animal or plant, it makes sense to also include the semantic categories ‘Animals’ with the semantic tag (AE) and ‘Plants’ with the semantic tag (AF). The semantic category AE contains 15 semantic tags, and the category AF contains 30 semantic tags.

#### 2.1.2. FoodOn Ontology

FoodOn [1] is a harmonized food ontology—a regulated vocabulary that can be used by humans and computers—that names all components of animals, plants and fungi that may be used as food for humans and domesticated animals, as well as derived food items and manufacturing processes. It was developed with the goal to increase global food traceability, quality control and data integration (1). FoodOn [9] is a consortium-led project that aims to create a complete and readily accessible ontology for food that accurately and consistently identifies foods that are common in cultures around the world. FoodOn [1,9] bridges the gap between food product terminology and food traceability, it describes animal and plant food sources, food categories and products, as well as other aspects such as preservation procedures, touch surfaces and packaging, with a focus on human and domesticated animal food. The origin of FoodOn’s vocabulary is LanguaL [16]. It was constructed by transforming LanguaL into a World Wide Web Consortium (W3C) OWL Ontology Language-formatted vocabulary. FoodOn [1,9] offers over 9600 generic food product categories.

#### 2.1.3. OntoFood Ontology

OntoFood is an ontology available at the BioPortal [10]. It is an ontology with Semantic Web Rule Language (SWRL) rules, and it is designed for representation of the nutrition for diabetic patients.

#### 2.1.4. SNOMED CT Ontology

SNOMED CT, or Systematized Nomenclature of Medicine—Clinical Terms [2], is a terminological resource that can have many purposes in applicative studies and software applications from the biomedical and health domain. It is standardized, available in many languages and widely used by healthcare providers and practitioners for electronic health records (EHRs). As a resource, SNOMED CT [2] can enable clinical record systems to incorporate clinical data entries, link them to knowledge bases, allow sophisticated analysis to be conducted on them, etc. SNOMED CT [2] is also part of the Unified Medical Language Systems (UMLS) [17] and is considered to be one of the most comprehensive healthcare terminologies; it lays the groundwork for electronic health records (EHR) across the clinical healthcare spectrum, from direct patient–practitioner contacts to laboratory testing and reporting, as well as statistical analysis. Out of our interest for this study, from the SNOMED CT [2] vocabulary of course is the Food concept, which exists in the resource besides the medical concepts which are the main focus.

#### 2.1.5. FoodEx2

FoodEx2 classification system—FoodEx2 is the second version of FoodEx [11]—is a comprehensive standardized system for food classification and description which aims to address the requirement to describe food in datasets across a variety of food safety domains. It was developed by the European Food Safety Authority (EFSA) [18], in 2011, and following its first release in 2011, the system was extensively tested in a variety of real-world scenarios, allowing for evaluation and the identification of areas for improvement. The system has domain knowledge embedded in it, and it contains descriptions of a vast set of individual food items combined in food groups and more broad food categories in a hierarchy that exhibits parent–child relationship.

#### 2.1.6. FoodBase

**Figure 1:**
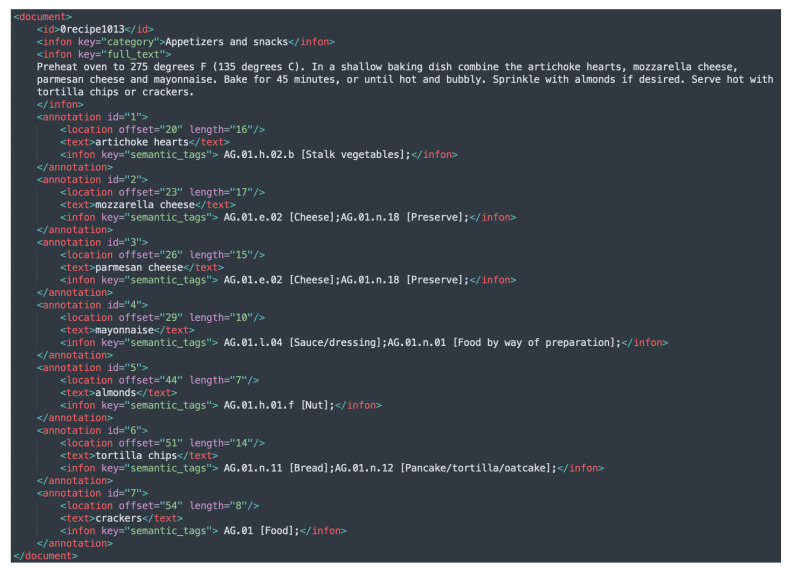
Example instance from the FoodBase-curated corpus.

FoodBase [6] is an annotated corpus of food entities, constructed using recipes extracted from Allrecipes (the largest social network for food related data) [19]. The recipes in FoodBase [6] are divided into five categories, and semantic tags from the Hansard corpus [3,7] are used for annotating the food entities. The corpus is divided into two sections: curated and uncurated. Both are developed using a rule-based named-entity recognition method known as FoodIE [5]. The curated version is checked and corrected by domain experts to guarantee that it contains ground-true annotations, whilst the uncurated version uses annotations directly provided by FoodIE, and it includes false positives and false negatives. The curated version of the FoodBase corpus [6] consists of 1000 recipes, whereas the uncurated has roughly 22,000 recipes. Each food entity retrieved using FoodIE is annotated using the semantic tags supplied by the Hansard corpus [3,7]. The extracted entities are food concepts annotated with the semantic tags that originate from three basic categories in the Hansard taxonomy: ‘Food and drink’, ‘Animals’ and ‘Plants’ (AF). The priority is to annotate the food chunks with semantic tags from the ‘Food and drink category’, but when no semantic tag from that category is identified, a tag from either ‘Animals’ or ‘Plants’ is utilized. Furthermore, if no semantic tags can be found for a food object, it is assigned to the top-food-level hierarchy, i.e., ‘AG.01 [Food]’. Figure 1 presents an example of an annotated recipe from the FoodBase corpus.

#### 2.1.7. FoodOntoMap

FoodOntoMap [12] is a resource for food concept mapping across different food semantic resources. The main purpose behind the development of the FoodOntoMap resource is to align food concepts in different food ontologies. The food concepts used are extracted from recipes and are the concepts available in the curated version of FoodBase [6]. Semantic tags from four different food semantic resources are applied to each extracted food concept. The resources for alignment chosen are: Hansard corpus [3,7], FoodOn [1,9], OntoFood [10] and SNOMED CT [2]. As a resource, FoodOntoMap connects, i.e., links, several food ontologies which can then be utilized to construct applications for better understanding the interconnection between food systems, human health and the environment. It consists of linking data that allows us to find the semantic tag for the same food concept in different semantic resources. For this purpose, for each semantic resource, the semantic tag for each unique food concept (i.e., described with its ID) is stored in a separate table, which is further linked in the linking data.

### 2.2. Annotations Methods

#### 2.2.1. NCBO Annotator

The NCBO annotator [13] is a Web service available in the BioPortal [8] software that annotates raw, unstructured text provided by the user, with concepts from ontologies chosen by the user. The annotation workflow is based on a syntactic concept recognition engine, using concept names and synonyms, and a collection of semantic extension algorithms that take advantage of the semantic in the chosen ontology/ontologies [13]. The technique uses ontologies to generate annotations on raw, unstructured text, which are then returned via semantic web standards. The NCBO annotator is used for the development of FoodOntoMap resource.

#### 2.2.2. StandFood

StandFood [14] is a semi-automated method for standardizing foods in accordance with the FoodEx2 classification system [11]. It is a lexical similarity method that, based on a probability that captures the morphological information in the food concept name, defines the most similar food concept that exists in the FoodEx2 system [11].

The original version of this method was written in *R* [20]; since its publication in 2017, some of the packages are outdated and the core of the method—the part-of-speech (POS) tagger—ceased to exist. Therefore, this method has been transferred to the latest version of Python, updated with state-of-the-art POS tagger [21].

#### 2.2.3. Annotating Food Consumption Data with Regard to Different Semantic Resources

**Figure 2:**
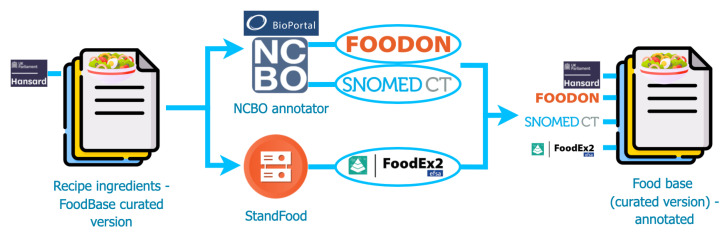
Flowchart of the methodology.

In Figure 2, we present a flowchart of the methodology used in this study. To provide annotated corpora with food entities with regard to different semantic resources that can further play an important role in developing NLP-based tools that can help different food safety applications, we propose a pipeline that consists of two different steps for annotations. The starting point is the gold standard of FoodBase [6], i.e., the FoodBase-curated version which contains 1000 manually evaluated recipes, annotated with the appropriate semantic tags from the Hansard taxonomy. Our goal is to make this corpus available with annotations from FoodON, SNOMED CT and with semi-automatic-assigned FoodEx2 codes. For annotations with regard to FoodOn [1,9] and SNOMED CT, we link the FoodBase corpus [6] using the FoodOntoMap [12] and the NCBO annotator [13], while for FoodEx2 [11] annotations, we use the StandFood [14] lexical similarity method.

1. Linking the FoodBase corpus with the FoodOntoMap resource—FoodOn and SNOMED CT annotations
  This linking was performed in a few steps:
  (a) Select all ingredients for each recipe from the curated version of FoodBase.
  (b) Use the NCBO annotator to obtain the semantic tags from FoodOn and SNOMED CT for each of the ingredients.
  (c) Assign the annotations obtained as a result from the NCBO annotator.
  (d) Using the BioC format, create new versions of the FoodBase-curated-version corpus:
    - FoodBase corpus with semantic tags from the FoodOn ontology.
    - FoodBase corpus with semantic tags from the SNOMED CT ontology.
2. Linking the FoodBase corpus using the StandFood method—FoodEx2 annotations
  This linking was performed in a few steps:
  (a) Select all ingredients for each recipe from the curated version of FoodBase.
  (b) Pre-process the text, i.e., the names of the ingredients, removing special characters, lemmatization, part-of-speech (POS) tagging [22].
  (c) Use StandFood to find the matches in the latest version of the FoodEx2 classification system.
  (d) Set a threshold for limiting the number of matches obtained; in our case, it was maximum of eight matches, as we determined that the probability after the eighth match was greatly decreased.
  (e) Assign the FoodEx2 codes of the matches to the ingredients.
  (f) Using the BioC format, create a new version of the FoodBase-curated-version corpus with the FoodEx2 codes assigned to each ingredient.

## 3. Results

In this study, we included 1000 instances of the FoodBase gold standard corpus [6]; therefore, the annotation process, both with the NCBO annotator through the BioPortal and the StandFood method with the help of Python libraries, provided the results relatively quickly (a matter of minutes). We should keep in mind that this process can be more lengthy depending on the size of the dataset of matter and the length of the phrases we are trying to annotate (for the StandFood method).

The result out of the annotation process with the NCBO annotator is a BioC XML format file, while from the StandFood method, we obtain a text file in a csv format (with the name of the food concept and the FoodEx2 matches—the number of which we previously specified), which is then converted to a BioC XML format as well. These BioC XML format files for each of the 1000 recipe instances from the curated FoodBase version contain:

- The full text of the recipe;
- A list of the annotations:
  - The ID of the annotation;
  - The text in the recipe representing the selected food concept;
  - The semantic tag/s from the semantic resource in question;
  - The location of the text representing the selected food concept (number of the beginning and ending characters).

Thus, the CafeteriaFCD is an annotated corpus of food consumption data with annotated food concepts from external semantic resources belonging to different domains, and it consists of four separate datasets available in the BioC XML format.

**Figure 3:**
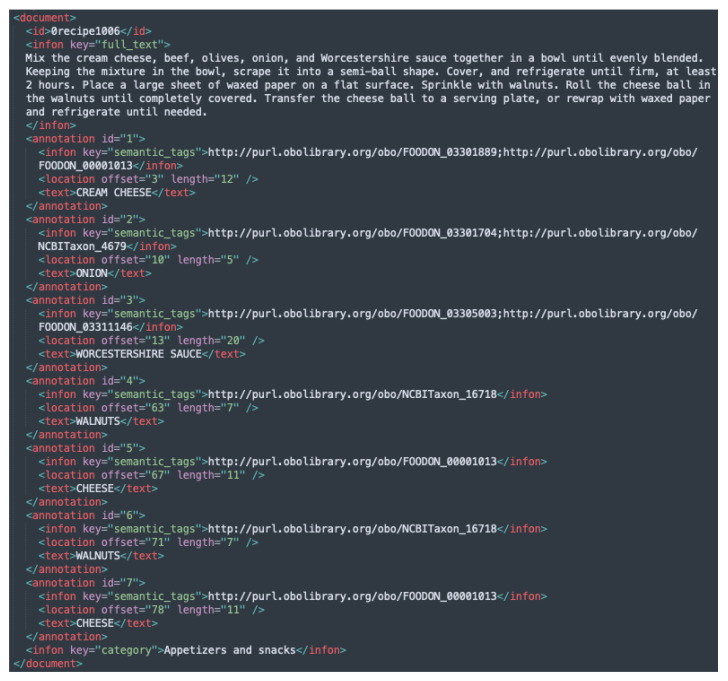
Example of an instance from FoodBase annotated with tags from the FoodOn ontology.

**Figure 4:**
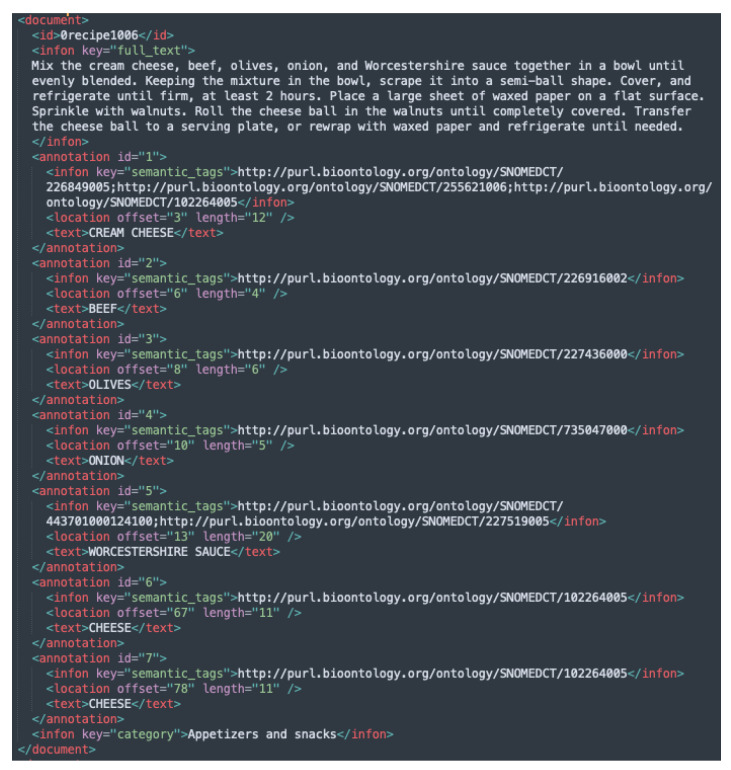
Example of an instance from FoodBase annotated with tags from the SNOMED CT ontology.

**Figure 5:**
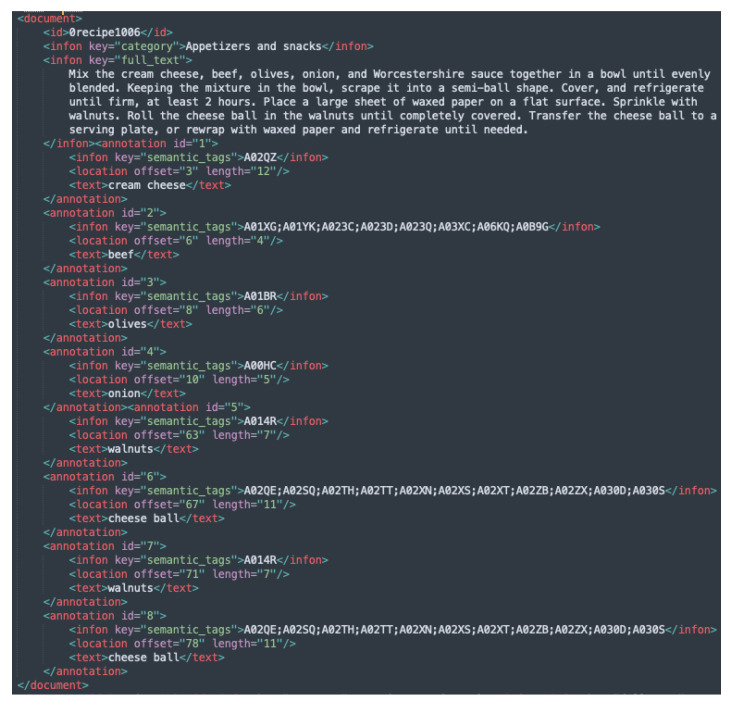
Example of an instance from FoodBase annotated with tags from the FoodEx2 classification system.

Example instances from each of the newly added BioC XML files are given in Figure 3, Figure 4 and Figure 5, while the initial version with the Hansard corpus tags is presented in Figure 1.

**Figure 6:**
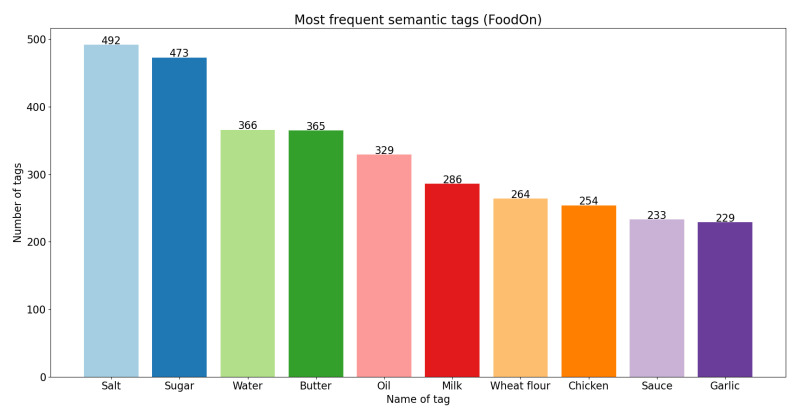
Ten most frequent semantic tags from FoodOn.

**Figure 7:**
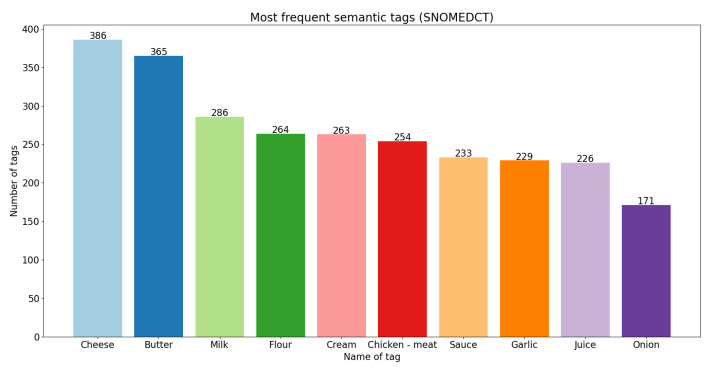
Ten most frequent semantic tags from SNOMED CT.

**Figure 8:**
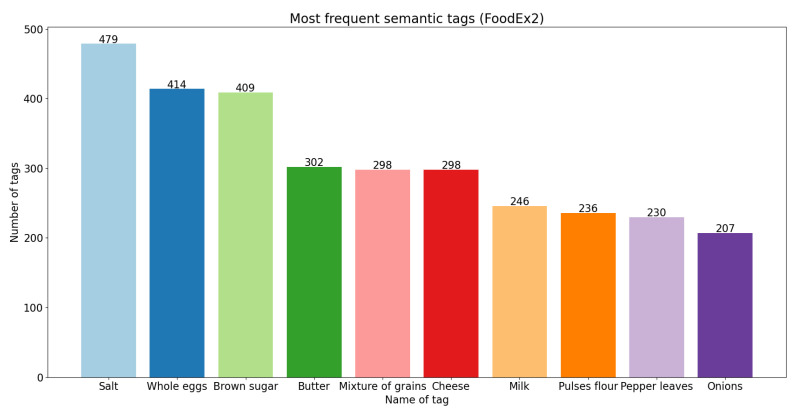
Ten most frequent semantic tags from FoodEx2.

On the other hand, in Figure 6, Figure 7 and Figure 8, we present the 10 most frequent semantic tags from each semantic resource and the number of food concepts annotated with these semantic tags.

From the bar plots, we can see that with FoodOn [1,9] we have the highest number of annotated instances. The tag ‘Salt’ appears as the number one most frequent from FoodEx2 and FoodOn; however, from the SNOMED CT ontology, it does not appear in the top 10 most frequent. We can also see tags with the same name that appear in all three bar plots: ‘Butter’, ‘Milk’, as well as tags that appear in two of the three plots, ‘Onion’, ‘Garlic’ and ‘Sauce’. There are tags for the same foods that are present with different names in the resources, such as: ‘Pulses flour’, ‘Flour’ and ‘Wheat flour’; ‘Chicken—meat’ and ‘Chicken’. Overall, we can say that the FoodOn [1,9] ontology provided the most expected results—giving the most generic and basic foods that are the most commonly used ingredients for recipes as the top 5 most frequent semantic tags: ‘Salt’, ‘Sugar’, ‘Water’, ‘Butter’ and ‘Oil’.

With regard to the semi-automatic annotation using the FoodEx2 system, out of the 10,719 entities/ingredients from the 1000 gold standard recipes in the FoodBase corpus, 9743 were annotated with one or more FoodEx2 codes, and for the 976 left, the StandFood method was not able to find any matches from the FoodEx2 dictionary. When the duplicates were removed, from the 976 entities that had no annotation assigned, only 266 were left, meaning there are 266 ingredients without annotations.

In order to improve the results, we decided to incorporate an assignment of the FoodEx2 codes based on the priority of three approaches:

1. The StandFood method—limiting the number of matches to eight;
2. String matching—matching the lemmas of the words in the names of the foods from the dataset and the names of the foods in the FoodEx2 dataset;
3. Character matching—calculating the string similarity based on the Levenshtein distance between the lemmas of the words in the names of the foods from the dataset and the names of the foods in the FoodEx2 dataset. We opted to use the Levenshtein distance because it is the most frequently used string similarity measure.

When the matching was performed in this way, we obtained a new dataset, where the number of unannotated instances was 0. Meaning all 10,719 entities from the FoodBase corpus [6] were annotated with a FoodEx2 code/tag. The descriptive statistics from Table 1 changed as well, and the new ones are presented in Table 2.

## 4. Discussion

The motivation behind this study was to contribute to the food and nutrition domain with a domain-specific semantic resource which can open annotate a corpus with food consumption data with semantic tags from external resources. The central resource is a product from another study [6]—the FoodBase corpus [6] is a corpus with recipe data, already annotated with semantic tags from the Hansard corpus [3,7]. Having this corpus, we presented the annotation process of food consumption data (recipes) with semantic tags from different semantic resources: Hansard taxonomy [3,7], FoodOn ontology [1,9], SNOMED CT [2] terminology and the FoodEx2 classification system [11]. Two different approaches for annotating were used for the different resources—as FoodOn [1,9] and SNOMED CT [2] are both ontologies and available at the BioPortal [8], the NCBO annotator [13] was used for the annotations. FoodEx2, on the other hand, is a coding system, and even though it is very useful and insightful, it is a little tricky to work with. As the most used method for annotating is manual domain-expert annotation, the only method for automating this process is the StandFood method, and that was what was used in order to obtain the FoodEx2 codes.

However, there is something that needs to be taken into account about the annotations with the FoodEx2 codes. The StandFood method is a semi-automatic method for annotation—as a result, it returns multiple choices (user specified) that then need to be checked by the domain experts. Having that in mind, the annotations obtained with regard to the FoodEx2 hierarchical classification system cannot fit the golden standard; therefore, they are assumed as a silver standard. On the contrary, the annotation process with regard to Hansard [3,7], FoodOn [1,9] and SNOMED CT [2] is fully automated and approved by domain experts; accordingly, it can be assumed as a gold standard.

After the annotation process, the end result is a new version of the golden standard of the FoodBase corpus, called CafeteriaFCD. This corpus contains semantic tags from all the above-mentioned semantic resources; therefore, with these annotations, all these resources are interlinked, and the data interoperability is made possible across a total of five semantic resources from various domains. This level of data comprehension and interoperability opens new opportunities for further utilizing this corpus for developing information extraction pipelines that can be further used for tracing knowledge about food safety applications.

Providing an annotated corpus with regard to different semantic resources allows us to further train information extraction methods using the state of the art in NLP. These resources were further utilized for training NER methods and their utility has already been published in [23].

## Figures and Tables

**Table 1:** Descriptive statistics about the FoodEx2 annotations on the FoodBase dataset.

Number of FoodEx2 Annotations	Total Number of Annotated Instances	Number of Annotated Instances (Without Duplicates)
1	5955	1076
2	801	234
3	621	91
4	300	64
5	146	36
6	358	58
7	192	58
8	1370	213

**Table 2:** Descriptive statistics about the FoodEx2 annotations on the FoodBase dataset.

Number of FoodEx2 Annotations	Total Number of Annotated Instances	Number of Annotated Instances (Without Duplicates)
1	5825	1070
2	1056	270
3	755	132
4	530	81
5	286	72
6	158	61
7	157	13
8	1953	407

## Data Availability

The corpora that are a product of this study are available on the following Zenodo repository: https://zenodo.org/record/6794907#.YsL9THZBzek (accessed on 4 July 2022).

## Abbreviations

The following abbreviations are used in this manuscript:

CafeteriaFCD	Cafeteria Food Consumption Data
IE	Information Extraction
NER	Named-Entity Recognition
NLP	Natural Language Processing
POS	Part-of-Speech Tagging
UMLS	Unified Medical Language System
